# Chronic Treatment with the AMP-Kinase Activator AICAR Increases Glycogen Storage and Fatty Acid Oxidation in Skeletal Muscles but Does Not Reduce Hyperglucagonemia and Hyperglycemia in Insulin Deficient Rats

**DOI:** 10.1371/journal.pone.0062190

**Published:** 2013-04-19

**Authors:** Kaio F. Vitzel, George Bikopoulos, Steven Hung, Kathryn E. Pistor, Jessica D. Patterson, Rui Curi, Rolando B. Ceddia

**Affiliations:** 1 Muscle Health Research Center – School of Kinesiology and Health Science, York University, Toronto, Ontario, Canada; 2 Department of Physiology and Biophysics, Institute of Biomedical Sciences, University of Sao Paulo, Sao Paulo, Brazil; INSERM/UMR 1048, France

## Abstract

This study tested whether the glycogen-accumulating effect of chronic *in vivo* pharmacological 5′AMP-activated protein kinase (AMPK) activation could improve glycemic control under conditions of insulin deficiency. Male Wistar rats were rendered diabetic through the administration of streptozotocin (STZ) and then treated for 7 consecutive days with the AMPK activator 5-aminoimidazole-4-carboxamide-1-β-D-ribofuranoside (AICAR). Subsequently, glycogen content and synthesis, glucose oxidation, and fatty acid oxidation (FAO) were determined in oxidative and glycolytic skeletal muscles. Glycemia, insulinemia, glucagonemia, and circulating triglycerides (TG) and non-esterified fatty acids (NEFAs) were measured after AICAR treatment. Insulin was almost undetectable in STZ rats and these animals were severely hyperglycemic. Glycogen content was markedly low mainly in glycolytic muscles of STZ rats and AICAR treatment restored it to control values. No differences were found among all muscles studied with regards to the content and phosphorylation of Akt/protein kinase B and glycogen synthase kinase 3. Even though glycogen synthase content was reduced in all muscles from STZ rats, insulin-induced dephosphorylation/activation of this enzyme was preserved and unaffected by AICAR treatment. Glucagon and NEFAS were 2- and 7.4-fold fold higher in STZ rats than controls, respectively. AICAR did not affect hyperglycemia and hyperglucagonemia in STZ rats; however, it normalized circulating NEFAs and significantly increased FAO in glycolytic muscles. In conclusion, even though AICAR-induced AMPK activation enhanced glycogen accumulation in glycolytic muscles and normalized circulating NEFAs and TG levels, the hyperglycemic effects of glucagon likely offset the potentially glucose-lowering effects of AICAR, resulting in no improvement of glycemic control in insulin-deficient rats.

## Introduction

Skeletal muscle accounts for ∼40% of total body mass in a reference adult male and ∼30% in a reference adult female [1] and has the capacity to store up to 1 to 2% of its weight in glycogen [2]. Furthermore, it has been estimated that muscle glycogen synthesis accounts for the majority of whole-body glucose uptake and virtually the entire nonoxidative glucose metabolism [3]. These features make the skeletal muscle a crucial compartment for the regulation and maintenance of whole-body glucose homeostasis. Conditions that limit glycogen synthesis in skeletal muscles are actually associated with hyperglycemia and other metabolic disorders typically found in diabetic patients [2]. Glucose uptake and glycogen synthesis in skeletal muscles are closely linked and tightly regulated processes that require the recruitment of glucose transporter 4 (GLUT4) from intracellular vesicular structures to the cell surface and activation of glycogen synthase (GS), respectively [4]. Insulin is well known for its ability to potently stimulate both processes in skeletal muscle cells [2], [4]. However, there are insulin-independent mechanisms that also promote the recruitment of GLUT4 to the plasma membrane (e.g. muscle contractions) and increase intracellular glucose availability and regulate glycogen synthesis [4].

The molecular mechanisms underlying the insulin-independent stimulation of glucose uptake and glycogen synthesis in skeletal muscles are of great therapeutic interest for diabetes mellitus. Of particular interest is the cellular energy sensor AMP-activated protein kinase (AMPK), which is a serine/threonine protein kinase composed of one catalytic (α) and two regulatory (β, γ) subunits. Multiple isoforms of the three subunits exist (α1, α2, β1, β2, γ1, γ2, and γ3) and are differentially expressed in rodent and human skeletal muscle depending on fiber type composition [5], [6]. In mammalian cells, AMPK is activated under conditions of metabolic stresses that increase intracellular AMP, ADP or Ca^2+^ [7]. The interaction of AMP with cystathionine β-synthase sequence motifs located in the γ-subunit causes allosteric activation of AMPK [7]. However, it is the phosphorylation by upstream kinases of Thr172 in the activating loop of the α catalytic subunit of AMPK that causes the most prominent increase in its kinase activity [7], [8]. Besides metabolic stress, various drugs and xenobiotics [9] also induce the phosphorylation and activation of AMPK through mechanisms involving alterations in intracellular AMP, ADP, and Ca^2+^ levels or by reactive oxygen species production and DNA damage [7]. A drug extensively used to study the effects of acute and chronic AMPK activation in skeletal muscles either *in vitro* or *in vivo* is the AMP analog AICAR, which induces AMPK activation without altering the intracellular AMP:ATP ratio [9]. In skeletal muscles, the effects of AICAR on glucose uptake, GS activity, and glycogen synthesis have been demonstrated to be dependent on AMPK activation [10]–[12].

In its activated state, AMPK switches on catabolic pathways that generate ATP while switching off biosynthetic pathways that consume ATP, which is consistent with a role for AMPK in maintaining cellular energy homeostasis. In this context, it was expected that the energy consuming glycogen synthesis pathway would be suppressed by AMPK activation. This was supported by early studies in cell-free assays demonstrating that AMPK phoshorylates the inhibitory site of GS [13], by studies reporting that GS activity was reduced in muscles acutely exposed *in vivo* and *in vitro* to AICAR [10], [14], [15], and by the demonstration that insulin-stimulated glycogen synthesis was inhibited in isolated rat muscles acutely treated with this pharmacological AMPK activator [16]. However, other studies in which rats were chronically treated with AICAR have reported that AMPK activation promoted glycogen accumulation rather than depletion in skeletal muscles [17]–[19]. These apparently contradictory observations suggested that the direct acute inhibitory effect of AMPK on GS activity was overridden by other molecular events triggered under conditions of chronic AMPK activation. In this context, it was proposed that glycogen accumulation in skeletal muscle after chronic *in vivo* AICAR treatment was due to the well-known effects of this drug to increase glucose uptake and not because of AMPK-induced alterations in glycogen synthase and glycogen phosphorylase [20]. Subsequent studies indeed provided compelling evidence that AMPK promotes glycogen accumulation in skeletal muscles by allosteric activation of GS, an effect mediated by increased glucose uptake that leads to a rise in intracellular glucose-6-phosphate (G6P) [12]. In this model, the allosteric activation of GS by elevated intracellular G6P overrides the potential inhibitory-effect that AMPK exerts on glycogen synthesis by phosphorylating GS [12], leading to increased glycogen content in skeletal muscles.

Previous studies have demonstrated that chronic AICAR-induced AMPK activation significantly increased glycogen synthesis in glycolytic muscles and improved glycemic control in insulin resistant rats [21], [22]; however, it is yet to be determined whether or not similar effects of chronic *in vivo* pharmacological AMPK activation could be achieved under conditions of insulin deficiency. In this study, we hypothesized that the insulin-independent effects of AMPK activation that promote glycogen accumulation in skeletal muscles could also improve glycemic control under conditions that insulin is lacking. This could be of therapeutic importance for type 1 diabetes. In order to test this hypothesis, rats were rendered diabetic through the administration of the pancreatic-β-cell-toxic drug streptozotocin [23]. Diabetic animals with almost undetectable levels of circulating insulin were then treated for 7 consecutive days with AICAR. Glycemia, insulinemia, glucagonemia, and levels of TG and NEFAs in the circulation were determined prior to and during the treatment. Since many of the metabolic effects of AICAR-induced AMPK activation on skeletal muscle have been reported to be fiber type-specific [16], [18], [19], [21], [24], FAO, glucose oxidation, glycogen synthesis and content were determined in oxidative and glycolytic skeletal muscles. Also, major signaling pathways involved in glycogen synthesis under basal and insulin-stimulated conditions were thoroughly assessed in these muscles. Our findings show that chronic pharmacological AMPK activation robustly increased glycogen content in glycolytic skeletal muscles in insulin-deficient rats. This was also accompanied by enhanced skeletal muscle FAO and normalization of NEFA and TG levels in the circulation. However, the severe hyperglycemia of diabetic rats chronically treated with AICAR was not improved.

## Materials and Methods

### Animals

Male albino rats from the Wistar strain (Charles River Laboratories, Montreal, Quebec, Canada) weighing 180–200 g (initial weight) were used in all experiments. The animals were housed in cages with free access to water and standard rat chow. The animals were maintained in a constant-temperature (23°C), with a fixed 12-h light, 12-h dark cycle (07∶00–19∶00 h). The protocol containing all animal procedures described in this study were specifically approved by the Committee on the Ethics of Animal Experiments of York University (York University Animal Care Committee, YUACC, permit number: 2011–14) and performed strictly in accordance with the YUACC guidelines. All surgery was performed under Ketamine/Xylazine anesthesia, and all efforts were made to minimize suffering.

### Reagents

AICAR was purchased from Toronto Research Chemicals (Toronto, Ontario). Amyloglucosidase, FA-free bovine serum albumin (BSA), glycogen, glucose-6-phosphate dehydrogenase, hexokinase, palmitic acid, and STZ were obtained from Sigma (St. Louis, MO). Human insulin (Humulin R) was purchased from Eli Lilly Inc. (Toronto, Ontario, Canada). ATP and nicotinamide adenine dinucleotide phosphate were obtained from BioShop Canada Inc. (Burlington, Ontario, Canada). D-[U-^14^C]glucose and [1-^14^C]palmitic acid was from GE Healthcare Radiochemicals (Quebec City, Quebec). Specific antibodies against Akt, P-Akt (Thr308 and Ser 473), GSK3, P-GSK-3α (Ser 21), GS, P-GS (Ser641, site 3a), AMPK, and P-AMPK (Thr172) were purchased from Cell Signaling Technology Inc. (Beverly, MA).

### Streptozotocin (STZ)-induced Diabetes and AICAR Treatment

Male Wistar rats (180–200 g) were fasted overnight and then rendered diabetic by a single intraperitoneal injection of STZ (65 mg/kg of body weight). After the STZ injection, animals had *ad libitum* access to food and water. Fourty eight hours after STZ injection, blood was drawn from the saphenous vein to measure glycemia using LifeScan OneTouch Ultra glucometer. Only animals eliciting plasma glucose levels equal or higher than 25 mmol/l were used in this study. This was confirmed by measuring glycemia twice a week in all animals. In order to investigate the effects of chronic pharmacological AMPK activation on skeletal muscle metabolism and glycemia, three weeks after STZ injection control and diabetic animals received daily single IP injections containing either AICAR (0.4 g/kg b.w.) or vehicle (saline) during 7 consecutive days. AICAR was always injected between 09∶30 and 10∶00 am. At the end of the AICAR-treatment period, the animals were anaesthetized for tissue extraction and then immediately euthanized. The epididymal fat pad was thoroughly dissected and used as an indicator of adiposity.

### Determination of Metabolites and Hormones in the Serum

Blood from all animals was collected by saphenous vein bleeding and the serum was used to determine insulin (ELISA kit from Millipore, Billerica, MA), NEFAs (NEFA kit from Wako Chemicals, Richmond, VA), triglycerides [kit from BioVision, Milpitas, CA], and glucagon (ELISA kit from Phoenix Pharmaceuticals Inc., Burlingame, CA). All procedures were performed according to instructions provided by the manufacturers of the kits.

### Muscle Isolation and Incubation

At the end of the AICAR treatment-period, all animals were anesthetized with a single ip injection of ketamine/xylazine (0.6 mg and 10 mg/100 g body weight, respectively). Subsequently soleus (SOL), extensor digitorum longus (EDL) and epitrochlearis (EPI) muscles were quickly extracted. These muscles were chosen because of their wide range of reported fiber-type distributions. The percentages of type I, type IIa, and type IIb in SOL, EDL, and EPI muscles are 84/16/0, 3/57/40 [25], and 15/20/65 [26], respectively. Three sets of muscle strips (18–22 mg) were mounted onto thin stainless steel wire clips to maintain optimal resting length, and immediately placed in plastic scintillation vials containing 2 ml of pre-gassed [30 min with O_2_:CO_2_-95∶5% (vol/vol)] Krebs-Ringer bicarbonate (KRB) buffer containing 4% fat-free BSA and 6 mM glucose. The vials were sealed with rubber stoppers and gasification was continued for the entire 1 h pre-incubation period. One set of muscles was then transferred to vials containing 2 ml of the same KRB buffer plus D-[U-^14^C]glucose (0.2 µCi/ml) and incubated under continuous gasification for one additional hour either in the absence or presence of insulin (100 nM) for the determination of glycogen synthesis [16]. For the assessment of glucose oxidation, a centered isolated well containing a loosely folded piece of filter paper moistened with 0.2 ml of 2-phenylethylamine/methanol (1∶1, v/v) was inserted into the flasks where the muscles were incubated. After the 1 h-incubation period, the muscles were removed and the media were acidified with 0.2 ml of H_2_SO_4_ (5N). The flasks were maintained sealed at 37°C for an additional 1 h for collection of the ^14^CO_2_ released. Subsequently, the filter papers were carefully removed and transferred to scintillation vials for radioactivity counting. Another set of muscles was incubated for 20 min either in the absence or presence of insulin (100 nM), and then quickly frozen in liquid nitrogen (N_2_) for subsequent determination of content and phosphorylation of Akt, GSK3, and GS.

### Measurement of Glycogen Synthesis and Content in Isolated Muscles

Glycogen synthesis was assessed by measuring the incorporation of D-[U-^14^C]glucose into glycogen as previously described [16]. Briefly, immediately after incubation muscle strips were quickly washed in ice-cold PBS, blotted on filter paper, frozen (N_2_), and digested in 0.5 ml of 1M KOH at 70°C for 1 h. Of the digested muscle solution, aliquots were taken for the determination of glycogen content and glycogen synthesis. Glycogen was precipitated overnight (−20°C) with 100% ethanol, resuspended in 0.5 ml of water, and its radioactivity was determined using a scintillation counter. For measurement of glycogen content, the pH of muscle digest was titrated to 4.8 before the addition of acetate buffer and 0.5 mg/ml amyloglucosidase. Subsequently, glycogen was hydrolyzed at 40°C for 2 h and glucose was analyzed enzymatically and the absorbance read at 340 nm [16].

### Measurement of Palmitate Oxidation in Muscle Homogenates

Oxidative capacity of SOL and EDL muscles was assessed as described by Baldwin et al [27] with few modifications. Briefly, for the preparation of homogenates, ∼100 mg of SOL and EDL muscles were thoroughly minced in 200 µL of ice-cold SETH buffer (300 mmol/l sucrose, 2 mmol/l EDTA, and 10 mmol/l Tris-HCl, pH 7.4). Additional SETH buffer was added to yield a 20-fold (wt/vol) diluted minced tissue sample. The solution was then homogenized in an ice-cold Potter-Elvehjen glass homogenizer (10–12 passes across ∼30 seconds). Subsequently, 400 µl of muscle homogenates were transferred to plastic scintillation vials containing 1.6 ml of the reaction mixture (150 mmol/l sucrose +5 mmol/l MgCl_2_+30 mmol/l KCl +30 mM potassium phosphate buffer +2 mmol/l EDTA +2 mmol/l ADP +15 mmol/l Tris-HCl +1% BSA +0.75 mmol/l Palmitate +1 mmol/l carnitine +0.025 mmol/l CoA, pH 7.4) containing 0.2 µCi/ml [1-^14^C]palmitic acid. Cold and labeled palmitate were complexed with fat-free BSA prior to adding to the reaction mixture. Palmitate oxidation by soleus and EDL homogenates was measured by the production of ^14^CO_2_ from [1-^14^C]palmitic acid. The flasks where tissue homogenates were incubated had a centered isolated well containing a loosely folded piece of filter paper moistened with 0.2 ml of 2-phenylethylamine/methanol (1∶1, v/v). After the 1 h-incubation period, the media were acidified with 0.2 ml of H_2_SO_4_ (5N), and the flasks were maintained sealed at 37°C for an additional 1 h for collection of the ^14^CO_2_ released. Subsequently, the filter papers were carefully removed and transferred to scintillation vials for radioactivity counting [16].

### Western Blotting Analysis of Content and Phosphorylation of AMPK, Akt, GSK3, and GS

Pieces of muscles and incubated muscle strips were homogenized in a buffer containing 25 mmol/l Tris-HCl and 25 mmol/l NaCl (pH 7.4), 1 mmol/l MgCl_2_, 2.7 mmol/l KCl, 1% Triton-X, and protease and phosphatase inhibitors (0.5 mmol/l Na_3_VO_4_, 1 mmol/l NaF, 1 µmol/l leupeptin, 1 µmol/l pepstatin, and 20 mmol/l PMSF). Muscle homogenates were centrifuged, the infranatant collected, and an aliquot was used to measure protein by the Bradford method. Samples were diluted 1∶1 (vol/vol) with 2×Laemmli sample buffer, heated to 95°C for 5 min, and subjected to SDS-PAGE. All primary antibodies were used in a dilution of 1∶1,000 except for P-AMPK (1∶500). Equal loading was confirmed by Ponceau staining of all membranes.

### Statistical Analysis

Data were pooled from two independent experiments (N = 8–10 animals per group). Results are presented as means ± SEM. One- or two-way ANOVA followed by Tukey’s multi-comparison post-hoc tests was used to assess differences among groups. Differences were considered statistically significant at P<0.05.

## Results

### Blood Glucose, Insulin, Glucagon, TG, and NEFAs

**Figure 1:**
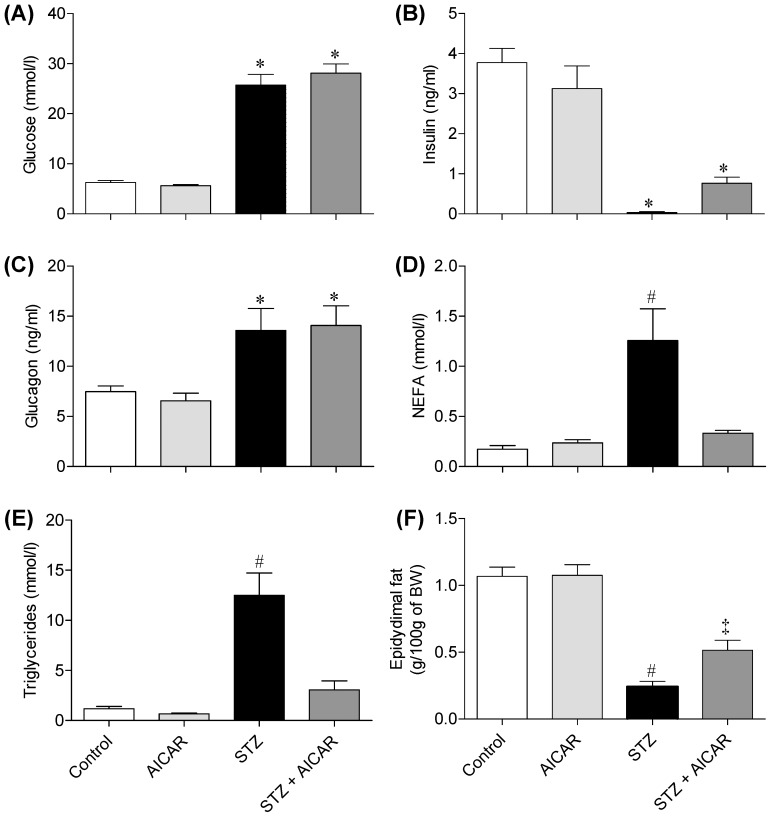
Effects of AICAR on blood glucose (A), insulin (B), glucagon (C), non-esterified fatty acids (NEFAs, D), tryglycerides E, and epidydimal fat mass (F). Blood and epidydimal fat were collected at the end of the study from control (saline-injected), AICAR, streptozotocin (STZ), and streptozotocin plus AICAR (STZ+AICAR) rats. Data from two sets of experiments were pooled together (N = 8–10 per group). *P<0.05 vs. control and AICAR. ^#^P<0.05 vs. control, AICAR, and STZ+AICAR. ^‡^P<0.05 vs. control, AICAR, and STZ (ANOVA).

In non-diabetic saline-injected rats (controls), blood glucose levels in the fed state remained constant (6.3±0.30 mmol/l) throughout the study. Similar values were found for non-diabetic AICAR-injected rats. In saline-injected STZ rats, blood glucose was 29.8±0.8 mmol/l and remained unchanged throughout the study, confirming the efficacy of STZ to induce diabetes in these animals. AICAR treatment did not change blood glucose levels in STZ rats (Figure 1A). Serum insulin did not differ between non-diabetic saline- and AICAR-injected rats. However, insulin was almost undetectable in the blood of STZ rats (Figure 1B), which is in line with the severe hyperglycemia found in these animals (Figure 1A). In AICAR-treated STZ rats, insulin levels were slightly higher than in STZ animals, although not reaching statistical significance (Figure 1B). While control and AICAR-treated non-diabetic rats had similar glucagon levels, STZ rats had values for this hormone that were almost 2-fold higher than controls. Treatment of STZ rats with AICAR did not affect circulating glucagon levels (Figure 1C). STZ rats had blood NEFA and TG levels 7.4-fold and 10.5-fold higher than control rats, respectively. Treatment of STZ rats with AICAR reduced circulating NEFAs and TG to values similar to those of non-diabetic saline-injected rats (Figure 1D and E).

### Epidydimal Fat Mass

While control and AICAR-treated rats had similar epidydimal fat pad masses, STZ rats had a marked reduction (85%) in this variable. Interestingly AICAR-treated STZ rats elicited a much lower reduction (57%) in epidydimal fat mass (Figure 1F). This indicates that AICAR significantly attenuated the reduction in fat mass observed in insulin-deficient rats, an effect that is compatible with the reduction in circulating NEFAs (Figure 1D) and TG (Figure 1E) found in AICAR-treated STZ rats.

### Glycogen Content in SOL, EDL, and EPI Muscles

**Figure 2:**
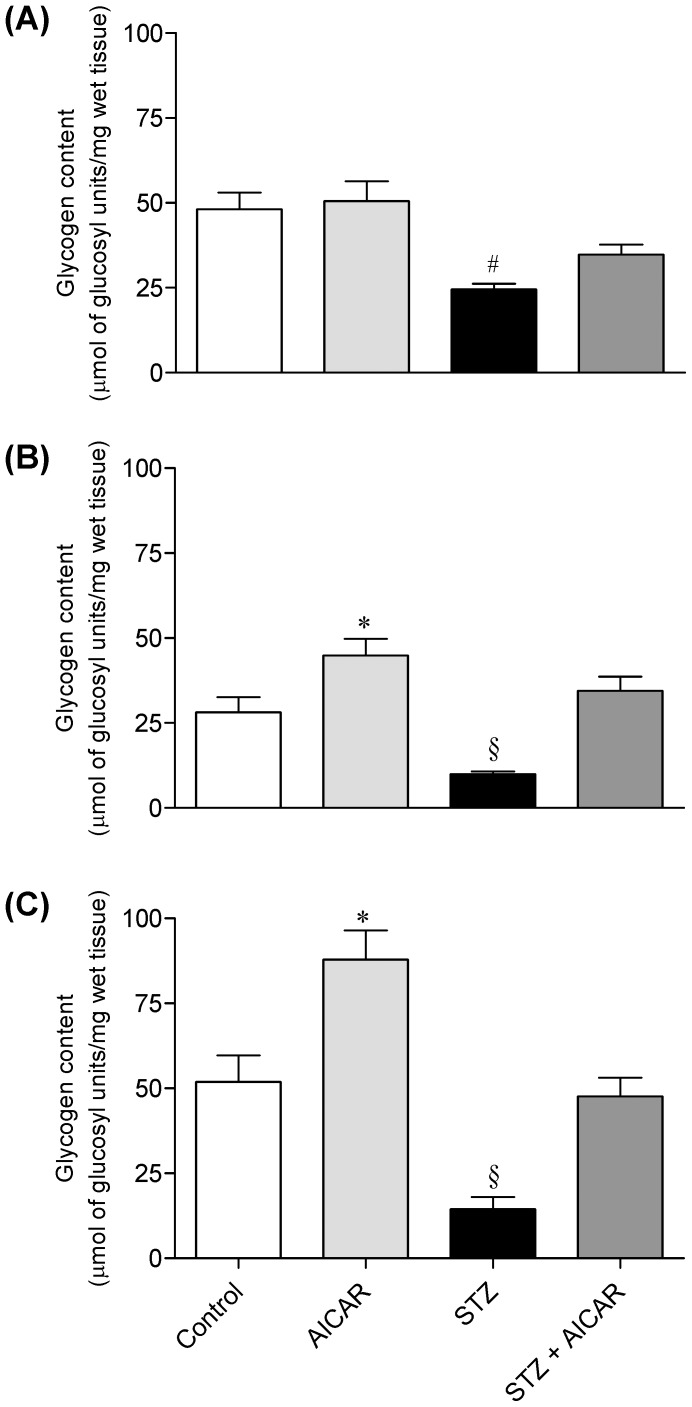
Effects of AICAR on glycogen content in soleus (A), EDL (B), and epitrochlearis (C) muscles. Muscles were isolated form control (saline-injected), AICAR, streptozotocin (STZ), and streptozotocin plus AICAR (STZ+AICAR) rats. *P<0.05 vs. control and AICAR. ^#^P<0.05 vs. all other groups (N = 6–8 per group, ANOVA).

No differences were found in glycogen content of the SOL muscles in non-diabetic control and AICAR-treated rats. In STZ rats; however, this variable was reduced by ∼50%. Even though glycogen content was slightly higher in SOL muscles of STZ rats treated with AICAR, this was not statistically different from STZ animals (Figure 2A). In EDL and EPI muscles of non-diabetic rats treated with AICAR, glycogen content was increased by 1.59- and 1.69-fold, respectively (Figure 2B and C). STZ rats had markedly lower glycogen content in EDL and EPI muscles than non-diabetic controls. AICAR treatment significantly increased glycogen content in these muscles, reaching values in EDL and EPI comparable to those of non-diabetic control rats (Figure 2B and C).

### Basal and Insulin-stimulated Glycogen Synthesis in SOL, EDL, and EPI Muscles

**Figure 3:**
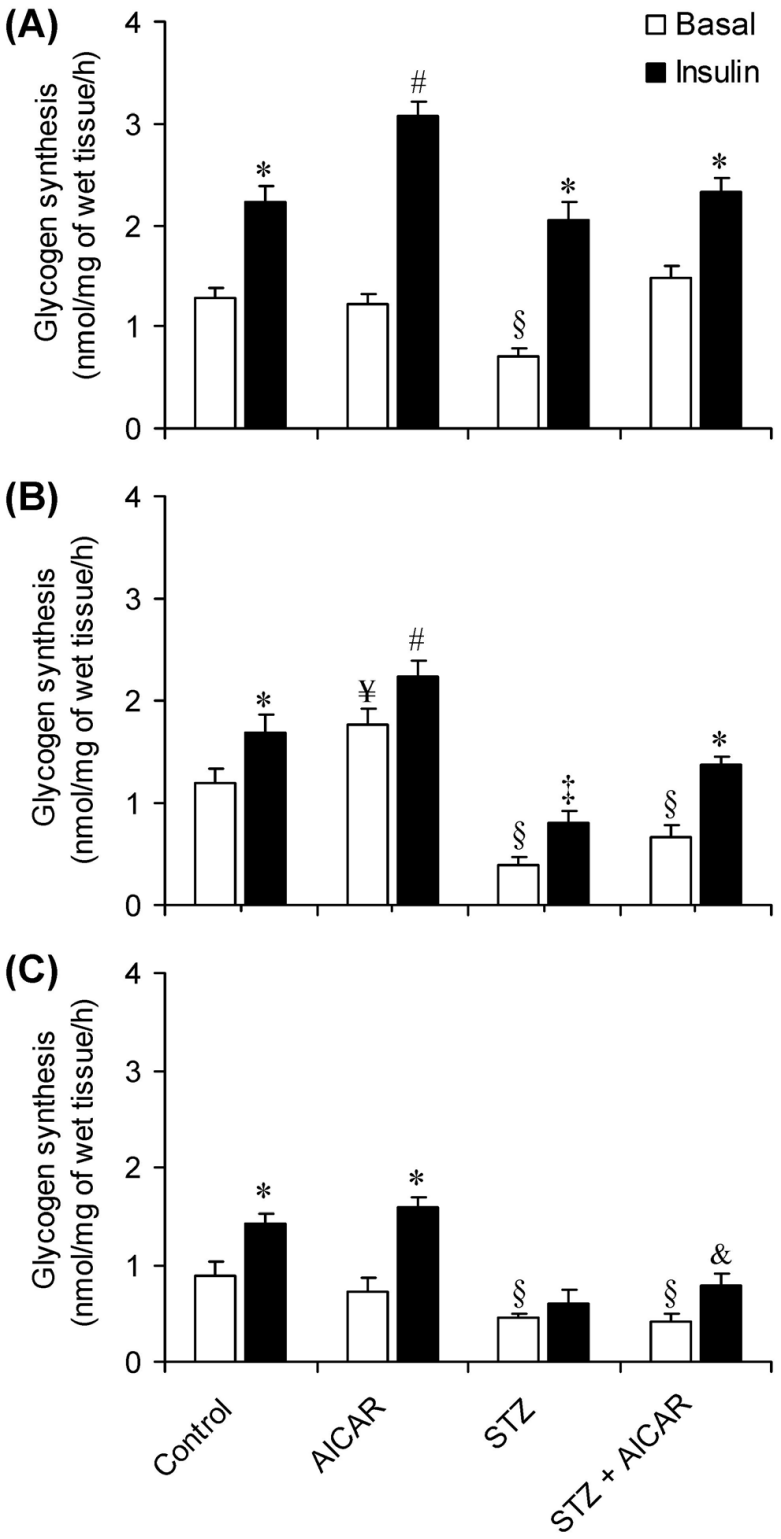
Effects of AICAR on basal (white bars) and insulin-stimulated (black bars) glycogen synthesis in soleus (A), EDL (B), and epitrochlearis (C) muscles isolated form control (saline-injected), AICAR, streptozotocin (STZ), and streptozotocin plus AICAR (STZ+AICAR) rats. Muscles were incubated for 1 h either in the absence or presence of insulin and assayed for the incorporation of ^14^C-glucose into glycogen. *P<0.05 vs. basal conditions; ^#^P<0.05 vs. all other groups; ^¥^P<0.05 vs. all basal conditions; ^§^P<0.05 vs. basal control and AICAR; ^&^P<0.05 vs. basal STZ and STZ+AICAR; ^‡^P<0.05 vs. basal STZ (N = 6–8 per group, Two-way ANOVA).

As expected, exposure to insulin increased glycogen synthesis by 1.76-fold (Figure 3A), 1.38-fold (Figure 3B), and 1.58-fold (Figure 3C) in SOL, EDL, and EPI muscles, respectively. In SOL and EDL muscles of non-diabetic rats, AICAR treatment potentiated the effect of insulin on glycogen synthesis and increased this variable by 1.37-fold (Figure 3A) and 1.33-fold (Figure 3B) when compared to non-diabetic controls, respectively. This effect was not observed in EPI, since the insulin-stimulated glycogen synthesis response of AICAR-treated non-diabetic muscles was similar to that of controls (Figure 3C). AICAR also significantly increased basal glycogen synthesis by 1.44-fold in EDL muscles when compared to controls (Figure 3B). Basal glycogen synthesis in SOL (Figure 3A), EDL (Figure 3B), and EPI (Figure 3C) muscles from STZ rats was significantly lower than control and AICAR-treated muscles. While the insulin-stimulated glycogen synthesis response was unaffected in SOL muscles of STZ rats, this variable was significantly lower than control for EDL (Figure 3B) and EPI (Figure 3C) muscles.

### Glucose Oxidation in SOL, EDL, and EPI Muscles

**Figure 4:**
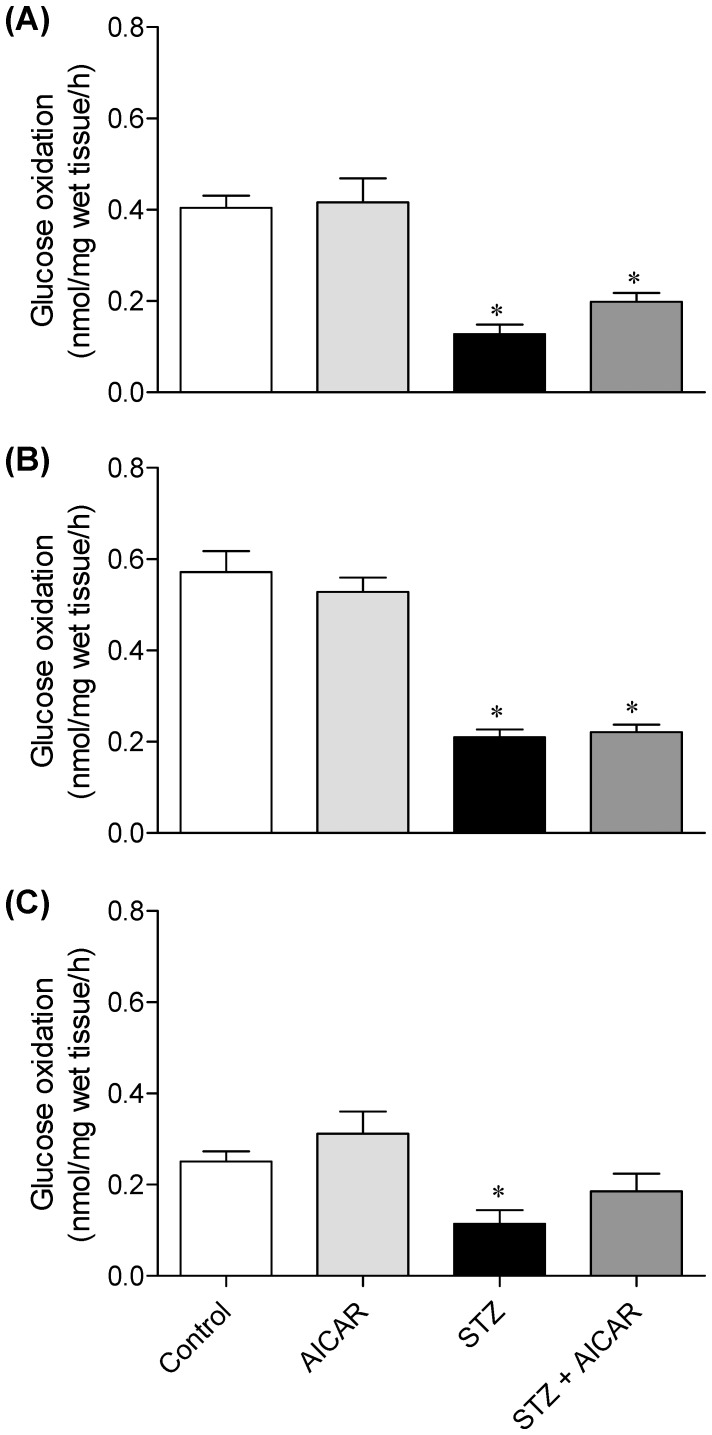
Effects of AICAR on glucose oxidation in soleus (A), EDL (B), and epitrochlearis (C) muscles. Muscles were isolated form control (saline-injected), AICAR, streptozotocin (STZ), and streptozotocin plus AICAR (STZ+AICAR) rats. *P<0.05 vs. control and AICAR (N = 6–8 per group, One-way ANOVA).

SOL, EDL, and EPI muscles from control and AICAR-treated rats elicited similar rates of glucose oxidation and this variable was significantly reduced by 56 to 67.5% in all three muscles from STZ rats. Treatment of STZ rats with AICAR did not affect glucose oxidation in any of the muscles studied (Figure 4A to C).

### Palmitate Oxidation in SOL and EDL Muscles

**Figure 5:**
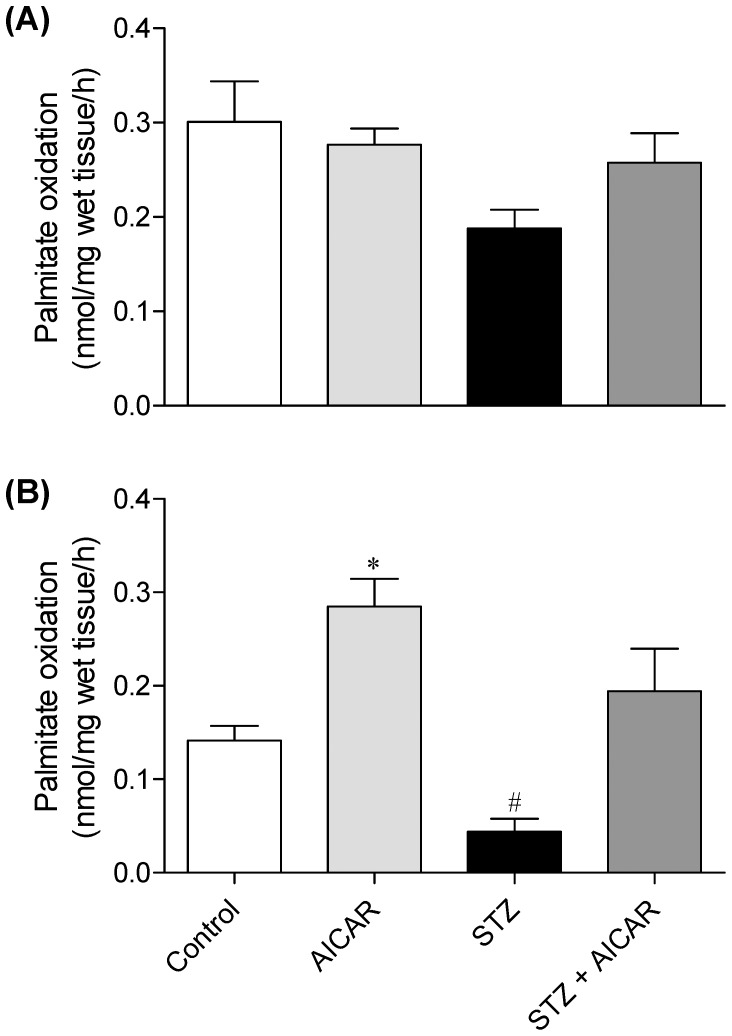
Effects of AICAR on palmitate oxidation in soleus (A) and extensor digitorum longus (B) muscles from control (saline-injected), AICAR, streptozotocin (STZ), and streptozotocin plus AICAR (STZ+AICAR) rats. *P<0.05 vs. control, STZ, and AICAR+STZ. ^#^P<0.05 vs. all other groups (N = 6–8 per group, One-way ANOVA).

Due to the extremely low capacity of EPI muscles to oxidize fatty acids found in previous studies conducted in our lab, in this study only SOL and EDL muscles were used for the measurement of palmitate oxidation. No significant differences were detected for palmitate oxidation in SOL muscles from control, AICAR, STZ, and STZ plus AICAR rats (Figure 5A). However, in EDL muscles from AICAR-treated rats palmitate oxidation was 2-fold higher than controls (Figure 5B). Furthermore, while in EDL muscles from STZ rats palmitate oxidation was reduced by almost 70%, AICAR treatment abolished this effect (Figure 5B).

### Phosphorylation and Content of AMPK, AKT, GSK3, and GS in SOL, EDL, and EPI Muscles

**Figure 6:**
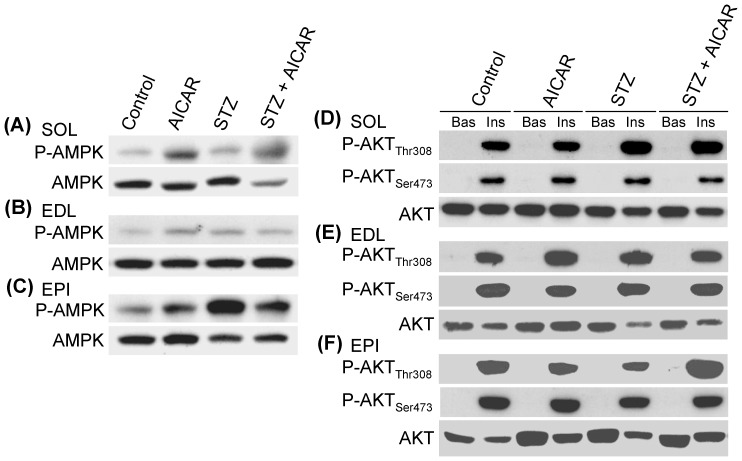
Representative blots of the effects of AICAR treatment on the content and phosphorylation of AMPK (A–C) and AKT (D–F) in soleus (SOL), extensor digitorum longus (EDL), and epitrochlearis (EPI) muscles of saline injected (control), AICAR, streptozotocin (STZ), and streptozotocin plus AICAR (STZ+AICAR) rats. AKT phosphorylation was determined in isolated muscles incubated for 20 min either under basal (Bas) or insulin-stimulated (Ins) conditions.

**Figure 7:**
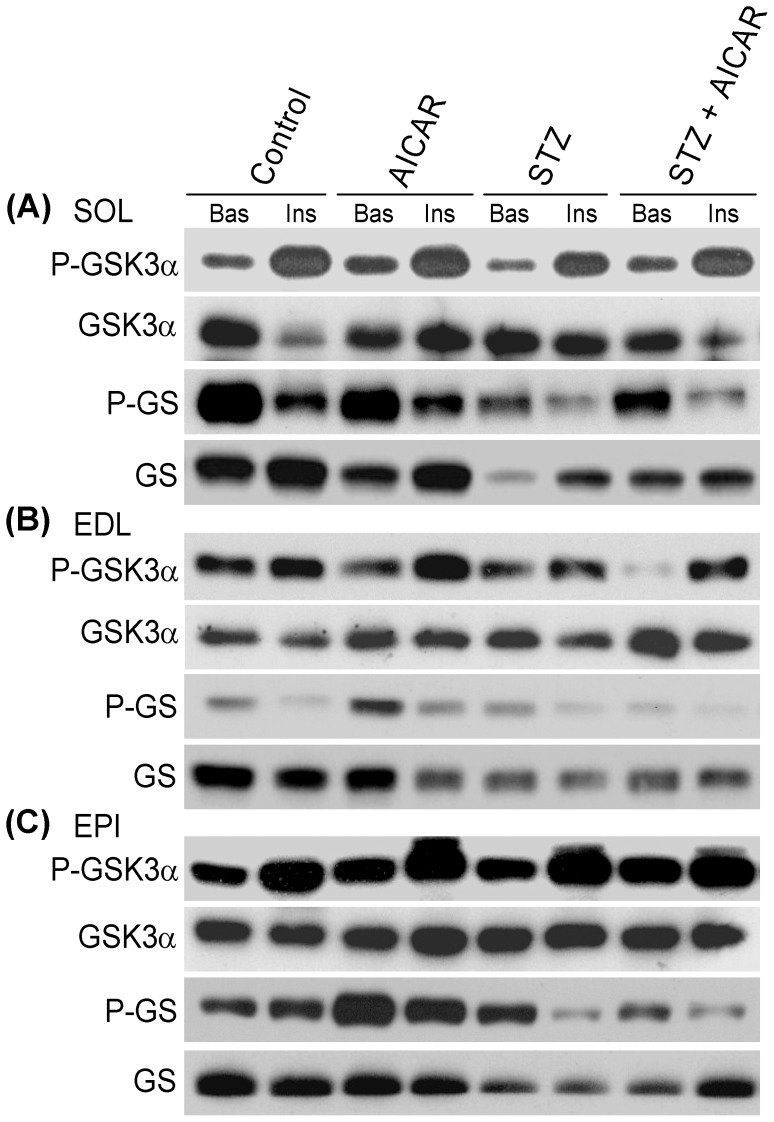
Representative blots of the effects of AICAR treatment on the content and phosphorylation of GSK3 and GS in soleus (SOL), extensor digitorum longus (EDL), and epitrochlearis (EPI) muscles of saline injected (control), AICAR, streptozotocin (STZ), and streptozotocin plus AICAR (STZ+AICAR) rats. GSK3 and GS phosphorylation was determined in isolated muscles incubated for 20 min either under basal (Bas) or insulin-stimulated (Ins) conditions.

As expected, AMPK phosphorylation was increased in SOL, EDL, and EPI muscles of non-diabetic AICAR-treated rats (Figure 6A–C). AMPK phosphorylation was also elevated in SOL, EDL, and EPI muscles of STZ rats. Treatment of STZ rats with AICAR actually attenuated AMPK phosphorylation in muscles from STZ rats. This effect seemed more pronounced in glycolytic muscles (particularly EPI) than oxidative (SOL) muscles (Figure 6A–C). AKT phosphorylation was undetected under basal conditions (Figure 6D–F). However, upon stimulation with insulin, the Thr308 and Ser473 residues of AKT were potently phosphorylated in isolated SOL, EDL, and EPI muscles from control, AICAR, STZ, and STZ plus AICAR rats (Figure 6D–F). No differences in AKT phosphorylation were detected between control and STZ rats either injected with saline or AICAR. Further analysis of GSK3α, a downstream target of AKT, revealed that under basal conditions phosphorylation of this protein was unaffected in SOL, EDL, and EPI muscles from control, AICAR, STZ, and STZ plus AICAR rats, respectively (Figure 7A–C). Upon stimulation with insulin, GSK3α phosphorylation was also potently increased in isolated SOL, EDL, and EPI muscles and no differences were found in this variable between control and diabetic rats either injected with saline or AICAR (Figure 7A–C). Under basal conditions, GS phosphorylation at Ser641 was much more pronounced in control and AICAR-treated than in STZ and STZ plus AICAR rats. This is likely attributed to a reduction in GS content observed in all muscles derived from STZ rats. However, despite the differences in muscle GS content between diabetic and non-diabetic rats, GS Ser641 phosphorylation was suppressed by insulin in isolated SOL, EDL, and EPI muscles from all groups of animals. Furthermore, prolonged AICAR treatment did not affect the ability of insulin to promote the suppression of GS phosphorylation in any of the muscles studied (Figure 7A–C).

## Discussion

Here, we provide novel evidence that chronic AICAR-induced AMPK activation *in vivo* did not reduce hyperglycemia in rats with STZ-induced diabetes, although it enhanced glycogen accumulation and fatty acid oxidation in white muscles and potently reduced circulating NEFA and TG levels in these animals. Insulin was almost undetectable in the blood of STZ rats and this was accompanied by marked reductions in glycogen content in SOL, EDL, and EPI muscles. Also, AMPK phosphorylation was increased in all muscles from STZ rats; however this response was more pronounced in EDL and EPI than SOL muscles. The lack of insulin clearly limited glucose availability to the muscle cells, a condition that on its own must have led to phosphorylation/activation of AMPK. In fact, previous studies have demonstrated that glucose deprivation is a powerful metabolic stress that stimulates AMPK activation in skeletal muscle cells [28], [29]. Interestingly, even though AMPK phosphorylation was higher in AICAR-treated rats, this variable seemed to have been attenuated by AICAR treatment, particularly in EPI muscles of STZ rats. The increase in glycogen content promoted by AICAR treatment seems to have minimized the stress of glucose deprivation in EPI muscles of STZ rats. Among the three muscles studied, EPI is the one with the lowest percentage of Type I and IIa fibers and elicits limited oxidative capacity, making it heavily dependent on glucose for energy production [26], [30]. In this study, EPI muscles of STZ rats treated with AICAR had a very robust increase in their glycogen contents after AICAR treatment, which is compatible with reduced metabolic stress and attenuation of AMPK phosphorylation. Our findings are consistent with previous observations that the effects of AICAR on glucose uptake and metabolism in skeletal muscles are fiber type-specific [16], [18], [19], [21], [24]. Despite having lower GLUT-4 protein content and glucose transport capacity than red, slow-twitch muscles [31], white, fast-twitch muscles elicited the most pronounced increase in glycogen content after chronic AICAR treatment [18], [19]. EDL and EPI muscles of STZ rats actually had the highest degree of glycogen depletion and were also the most responsive to AICAR, having their glycogen contents increased by AICAR treatment to values similar to those of control rats. The fiber type-specific response to AICAR was also noticed with respect to fatty acid oxidation, since EDL muscles robustly increased palmitate oxidation after AICAR treatment while SOL did not. This is in line with previous observations that AICAR increases both glycogen synthesis and fatty acid oxidation in white but not in red skeletal muscles *in vivo* [21].

Because glycogen content is dependent on the ability of the muscle to increase its rate of synthesis, we assessed the rate of glucose incorporation into glycogen in isolated SOL, EDL, and EPI muscles. This revealed that glycogen synthesis was mostly reduced in EDL and EPI muscles of STZ rats either under basal or insulin-stimulated conditions, which is compatible with the significant drop in the content of glycogen of these muscles. SOL muscles of STZ rats, on the other hand, had only their basal rates of glycogen synthesis reduced, which was then restored to control values upon AICAR treatment. In EDL and EPI muscles under basal conditions, AICAR treatment did not enhance the severely diminished rates of glycogen synthesis in STZ rats. In fact, only the insulin-stimulated responses of EDL and EPI were significantly increased in muscles from AICAR-treated STZ rats. Importantly, none of these effects could be attributed to alterations in signaling at the level of Akt and GSK3α, since no differences were found among all muscles studied with regards to the content and phosphorylation of these proteins either under basal or insulin-stimulated conditions. However, a marked reduction in the content of GS, a downstream target of GSK3 and the rate limiting enzyme for glycogen synthesis, was observed in SOL, EDL, and EPI muscles. Importantly, despite the reduction in GS content in all three muscles, the ability of insulin to induce dephosphorylation/activation of this enzyme was preserved and unaffected in STZ rats treated with AICAR. In this scenario, the low GS content must have limited the ability of the muscle cell to synthesize glycogen, which at least partially explains the reduced content of glycogen found in SOL, EDL, and EPI muscles of STZ rats. However, the reduced GS content is at odds with the significant increase in glycogen content found in EDL and EPI muscles of AICAR-treated STZ rats. Also, it is incompatible with previous demonstrations that AMPK activation phosphorylates sites 2 and 2a of GS and significantly inhibits its activity in glycogen depleted rat muscles [10]. What seems to reconcile these apparent discrepancies are the previous observations that chronic AICAR treatment promoted glycogen accumulation in rat skeletal muscle *in vivo* not by directly altering glycogen synthase and glycogen phosphorylase activities, but through the well known effect of AICAR to increase glucose uptake [20]. In fact, evidence has been provided that AICAR-induced AMPK activation elevates glycogen content by increasing glucose uptake and the intracellular availability of glucose-6-phosphate (G6P) [12]. As previously mentioned, the latter leads to allosteric activation of GS, which overrides the potential direct inhibitory effect of AICAR-induced AMPK activation on GS activity. This was elegantly demonstrated in a study where AICAR-induced glycogen synthesis was completely abolished in EDL muscles of mice expressing a mutated form of GS that could not be activated by G6P [12]. This could explain the findings of our study, particularly because glucose oxidation was markedly reduced in all muscles from STZ rats, and remained reduced even after AICAR treatment. In this context, more substrate could be diverted towards glycogen synthesis in a condition in which AICAR potentially increased both glucose uptake and G6P availability in skeletal muscles. Since STZ rats were treated with AICAR for 7 consecutive days, it allowed for the progressive build up of muscle glycogen in STZ rats even under conditions of reduced GS content.

In the present study, we also found that chronic AICAR treatment reduced the markedly elevated levels of circulating NEFAs in STZ rats to values similar to those of control animals. The AICAR-induced increase in skeletal muscle fatty acid oxidation found in this study and also reported by others [19], [21], [32], [33] certainly contributed to this effect. However, it is likely that NEFAs in the circulation were also lowered as a consequence of the inhibitory effect of AICAR on adipose tissue lipolysis [34], [35]. These are consistent with our findings that the drastic reduction in epidydimal fat mass observed in insulin-deficient STZ rats was significantly attenuated by AICAR treatment. The AICAR-induced increase in glycogen content and fatty acid oxidation in skeletal muscle and the reduction in circulating NEFAs and TG have been associated, either alone or in combination, with improved insulin sensitivity and reduced glycemia in normal rats [35], high-fat fed insulin resistant rats [21], and the ZDF leptin-receptor-deficient diabetes-prone rat [22], [36], [37]. In this study, we anticipated that chronic AICAR treatment would also reduce glycemia in the insulin deficient STZ rats. However, this was not the case, since STZ rats remained severely hyperglycemic after AICAR treatment. It is possible that under conditions of insulin deficiency the potential systemic glucose-lowering effect of AICAR was overridden by the effects of other abnormally altered gluco-regulatory hormones. In fact, we found that circulating glucagon was 2-fold higher in STZ rats than controls and this remained unaltered after STZ rats were treated with AICAR for 7 days. Hyperglucagonemia likely played an important role in preventing AICAR from reducing glycemia in STZ rats. Recent studies have indeed demonstrated that the elimination of glucagon action by whole-body deletion of its receptors prevents hyperglycemia in mice with STZ-induced insulin deficiency [38]. As previously mentioned, the hyperglycemic hepatic glycogenolytic and gluconeogenic effects of glucagon [39] likely offset the potentially glucose-lowering effect that AICAR exerted by increasing glycogen accumulation in skeletal muscles. Hence, it is possible that in a condition in which hyperglucagonemia is prevented; pharmacological AMPK activation may effectively reduce glycemia under conditions of insulin deficiency.
